# Nanotopography-Mediated Mechanotransduction Enhances hBMSCs Adhesion on TiO_2_ Nanotubes

**DOI:** 10.3390/jfb17040200

**Published:** 2026-04-19

**Authors:** Chenao Xiong, Hui Feng, Liyang Lu, Zehao Jing, Youhao Wang, Yiyuan Yang, Dexuan Meng, Yichen Zhang, Weishi Li, Hong Cai

**Affiliations:** 1 Engineering Research Center of Bone and Joint Precision Medicine, Beijing Key Laboratory of Advanced Bioadaptable Orthopedic Implants, Department of Orthopaedics, Peking University Third Hospital, Beijing 100191, China; xiongca@pku.edu.cn (C.X.); fenghuibjmu@bjmu.edu.cn (H.F.); jingzehao@bjmu.edu.cn (Z.J.); 2311210385@stu.pku.edu.cn (Y.W.); yangyiyuan@bjmu.edu.cn (Y.Y.); 2111210381@stu.pku.edu.cn (D.M.); 2411210441@pku.edu.cn (Y.Z.); 2 Peking University Aerospace School of Clinical Medicine, Beijing 100049, China; lly1996@pku.edu.cn

**Keywords:** nanotopography, titanium dioxide nanotubes, hBMSCs, proteomics, cell adhesion, mechanotransduction

## Abstract

Titanium and its alloys are widely used for orthopedic implants, but their intrinsic bioinertness may hinder osseointegration. In this study, titanium dioxide nanotube (TNT) arrays were fabricated on Ti-6Al-4V scaffolds via anodization, and their effects on the adhesion behavior of human bone marrow mesenchymal stem cells (hBMSCs) were investigated. Surface characterization showed that anodization successfully generated ordered TNT layers, increased surface roughness, enhanced protein adsorption, and induced an apparent superhydrophilic wetting response. Compared to the untreated scaffold and TNT50, the small-diameter TNT10 surface significantly promoted hBMSC adhesion and proliferation. Microscope imaging further revealed enhanced cell spreading, F-actin organization, and vinculin expression on TNT surfaces, with the most prominent focal adhesion-related staining observed in TNT10. Quantitative proteomic analysis showed that TNT10 was associated with coordinated remodeling of adhesion- and cytoskeleton-related molecular programs, including focal adhesion, cell–substrate junction, and regulation of the actin cytoskeleton. In contrast, TNT50, despite supporting obvious cytoskeletal remodeling, was more compatible with a dynamic, higher-turnover adhesion state. Overall, these findings suggest that small-diameter TNTs provide a more favorable interfacial microenvironment for stable early hBMSC adhesion on porous titanium scaffolds.

## 1. Introduction

Titanium and its alloys are among the most commonly used metallic materials for orthopedic implants, owing to their superior mechanical properties, corrosion resistance, and good biocompatibility [1]. Nevertheless, their intrinsic bioinertness may still limit rapid and stable osseointegration, especially during the early healing stage, making surface modification a widely adopted strategy to improve implant bioactivity [2]. hBMSCs are key progenitor cells involved in peri-implant bone regeneration. Their early recruitment and adhesion to the implant surface, followed by osteogenic differentiation, are essential prerequisites for initiating the osseointegration process [3,4]. Thus, optimizing material interface properties through surface engineering to enhance effective hBMSC adhesion has become a research focus in orthopedic biomaterials.

Recent studies show that nanoscale surface topography can effectively regulate cellular behavior and stem cell fate [5,6,7]. As an important physical microenvironmental cue, nanotopography modulates cell adhesion, migration, and lineage differentiation without the need for exogenous chemical factors [8,9]. Among various surface modifications, TNT arrays prepared via anodization on titanium surfaces have attracted extensive attention due to their structural controllability, reproducibility, and good biocompatibility [10,11]. Previous studies demonstrate that TNTs with specific geometrical features can modulate protein adsorption, cell attachment, spreading, proliferation, and osteogenic differentiation [12]. However, the reported cellular responses to TNT dimensions are not entirely consistent across different studies, indicating that the biological effects of nanotopography are governed by a complex interplay of multiple factors rather than by a single structural parameter alone [13,14].

At the cell–material interface, cells sense nanoscale topographical features primarily through transmembrane integrin receptors and integrin-associated focal adhesion complexes, which convert external physical cues into intracellular biochemical signals via mechanotransduction [15,16]. However, the molecular mechanisms by which cells undergo systemic proteomic remodeling to respond to specific nanotopographical cues and form stable adhesion complexes need further clarification.

To address these issues, in the present study, TNT arrays with different dimensions were fabricated on Ti-6Al-4V scaffolds via anodization. The scaffolds were co-cultured with hBMSCs in vitro to systematically evaluate the effects of TNT nanotopography on cell adhesion behavior and cytoskeletal remodeling. In addition, quantitative proteomic analysis was performed to explore the potential molecular mechanisms underlying nanotopography-regulated hBMSC adhesion from the perspective of global protein network remodeling. This study aims to provide further insight into the interaction between TNT-based nanotopography and stem cell adhesion, and to offer a theoretical basis for the rational design of titanium implant surfaces with improved osteointegration performance.

## 2. Materials and Methods

### 2.1. Material Preparation

The porous titanium alloy scaffold (TS) used in this study were provided by AK Medical (Beijing, China). The scaffolds were manufactured using Arcam Ti-6Al-4V standard powder via electron beam melting technology, with an internal diamond lattice structure. The core topological parameters included a pore size of 640 μm and a strut diameter of 400 μm. According to experimental requirements, two types of cylindrical samples were prepared: Φ10 mm × 5 mm for material characterization and in vitro cell assays, and Φ30 mm × 1 mm for proteomics studies.

Prior to anodization, the samples were ultrasonically cleaned in analytical-grade acetone, absolute ethanol, and deionized water for 15 min each, and then dried in a forced-air oven at 50 °C. The anodization electrolyte consisted of 97 vol% ethylene glycol, 3 vol% deionized water, and 0.5 wt% NH4F in a total volume of 250 mL, which is a commonly used electrolyte system for the fabrication of TNT arrays [17]. Anodization was performed under constant direct current voltages of 10 V or 50 V for 100 min. After the reaction, the samples were rinsed with deionized water, ultrasonically cleaned in ethylene glycol for 10 min, and thoroughly washed again with deionized water to remove residual electrolyte. The cleaned samples were dried and stored in a glass desiccator. Prior to biological experiments, the samples were sealed in radiation-permeable medical packaging and sterilized via γ-irradiation at a target absorbed dose of 25 kGy, followed by vacuum sealing and storage at room temperature in the dark.

### 2.2. Material Characterization

Samples were sputter-coated with gold using an ion sputter coater (Leica, Wetzlar, Germany) to improve surface conductivity. Surface morphology was examined via scanning electron microscopy (SEM, TESCAN, Brno, Czech Republic). Five randomly selected fields of view were recorded for each sample and used for subsequent measurements of nanotube diameter and length. Elemental composition was analyzed simultaneously via energy-dispersive X-ray spectroscopy (EDS). Three representative regions were randomly selected from each sample, and the average values of elemental weight percentage and atomic percentage are reported.

Three-dimensional surface topography and roughness were characterized using atomic force microscopy (AFM; Dimension Edge, Bruker, Billerica, MA, USA) in tapping mode with a scan area of 5 μm × 5 μm and a scan rate of 2 Hz. Three random regions were measured for each sample, and the corresponding roughness parameters were averaged (Ra, arithmetic average roughness; Rq, root mean square roughness; SAD, surface area difference).

Nanotube formation efficiency was quantified from SEM images using Amira software v2025.1 (Thermo Scientific, Waltham, MA, USA) through automated image analysis.

Surface wettability was evaluated via static water contact angle measurement using a contact angle goniometer (Krüss, Hamburg, Germany). Deionized water droplets (5 μL) were placed on the sample surfaces at room temperature (25 ± 1 °C), and the droplet morphology was recorded at 1 s intervals. The contact angle was measured automatically 30 s after droplet deposition, when the droplet profile had stabilized.

### 2.3. Protein Adsorption Assay

Scaffold samples (Φ10 mm × 5 mm) were placed in 24-well plates, and 1 mL of fetal bovine serum (FBS) working solution was added to each well. The working solution was prepared with 10% FBS (HyClone, Logan, UT, USA) in phosphate-buffered saline (PBS). Wells without scaffolds were used as blank controls. After incubation for 24 h at 37 °C, the solution in each well was gently mixed by pipetting and collected, followed by centrifugation at 500× *g* for 5 min. The resulting supernatant was transferred to fresh microcentrifuge tubes. After serial dilution, the total protein concentration and fibronectin (FN) concentration were measured using a bicinchoninic acid (BCA) protein assay kit (Beyotime, Shanghai, China) and a bovine FN ELISA kit (Jinclone, Beijing, China), respectively. The amount of protein adsorbed onto the scaffold surface was calculated as the difference between the protein content in the blank control and the corresponding sample group. Five parallel samples were tested in each group.

### 2.4. Cell Culture

hBMSCs (iCell, Shanghai, China) were cultured in α-MEM (HyClone, Logan, UT, USA) supplemented with 10% FBS (HyClone, USA) and 1% penicillin–streptomycin (100 U/mL penicillin and 0.1 mg/mL streptomycin). Cells were maintained in a humidified incubator at 37 °C with 5% CO_2_, and the culture medium was replaced every 2 days. When cell confluence reached approximately 80%, the cells were detached with 0.25% trypsin–EDTA (HyClone, USA) and passaged for subsequent experiments.

### 2.5. Cell Adhesion Assay

Scaffold samples (Φ10 mm × 5 mm) were placed in 24-well plates, and a cell suspension was seeded at a density of 2 × 10^5^ cells/mL (1 mL per well). After culturing for 6 or 24 h, the samples were transferred to a new plate and washed three times with PBS to remove non-adherent cells. Trypsin-EDTA was added and incubated at room temperature for 3 min to detach the adherent cells, and complete medium was then added to terminate the digestion. The cell suspensions were collected, and the number of adherent cells on each sample was counted using an automated cell counter (LUNA™, Anyang-si, Republic of Korea). Five parallel replicates were set for each group.

### 2.6. Cell Proliferation Evaluation

Scaffold samples (Φ10 mm × 5 mm) were placed in 24-well plates, and a cell suspension was seeded at a density of 1 × 10^5^ cells/mL (1 mL per well). After culturing for 24 h to ensure sufficient cell adhesion, the samples were transferred to a new plate, washed with PBS, and further cultured in complete medium. On days 3, 7, and 14 of culturing, the samples were transferred to a new plate, washed with PBS, and incubated with 1 mL cell counting kit-8 (CCK-8) working solution for 3 h at 37 °C in the dark. Subsequently, 100 μL of the reaction solution was transferred to a 96-well plate, and the optical density (OD) was measured at 450 nm using a microplate reader. Five parallel samples were tested per group.

### 2.7. Cell Morphology and Focal Adhesion Staining

Scaffold samples (Φ10 mm × 5 mm) were placed in 24-well plates, and cells were seeded at a density of 1 × 10^5^ cells/mL (1 mL per well) and cultured for 24 h.

For SEM observation, the samples were fixed with 2.5% glutaraldehyde for 1 h, and then dehydrated through a graded ethanol series (30%, 50%, 70%, 80%, 90%, and twice with 100% ethanol, 15 min for each concentration). The dehydrated samples were dried overnight in the dark at room temperature, sputter-coated with gold, and observed under SEM to evaluate cell spreading and filopodia extension on the scaffold surface. In addition, EDS coupled with SEM was employed for elemental analysis, focusing primarily on Ti and C elements, which enhanced the contrast between the titanium alloy substrate and the attached cells, resulting in clearer and more visually distinguishable images.

For cytoskeletal and focal adhesion staining, the samples were fixed with 4% paraformaldehyde at room temperature for 15 min, permeabilized with 0.1% Triton X-100 for 10 min, and blocked with 5% BSA blocking solution for 2 h. The scaffolds were then incubated with mouse anti-vinculin primary antibody (1:100) overnight at 4 °C, followed by incubation with Alexa Fluor 488-conjugated goat anti-mouse IgG secondary antibody (1:500) for 2 h at room temperature in the dark. Cell nuclei and F-actin were stained with DAPI (Solarbio, Beijng, China) and rhodamine phalloidin (Sigma, St. Louis, MO, USA) for 30 min each in the dark. Finally, images were acquired using a confocal laser scanning microscope (CLSM) to evaluate focal adhesion formation and cytoskeletal organization.

### 2.8. Proteomics-Based Adhesion Mechanism Research

Scaffold samples (Φ30 mm × 1 mm) were placed in 6-well plates, and a cell suspension was seeded at a density of 1 × 10^5^ cells/mL (200 μL was initially added dropwise to each well to promote early adhesion, followed by the addition of 2 mL medium). After 24 h of co-culture, the supernatant was discarded, and the samples were washed three times with pre-cooled PBS. Cells on the sample surfaces were scraped into centrifuge tubes using a cell scraper and pelleted at 1000 rpm for 2 min at 4 °C. RIPA lysis buffer containing protease and phosphatase inhibitors was added for 10 min of ice lysis, followed by centrifugation at 15,000 rpm for 10 min at 4 °C to collect the supernatant. The protein concentration was quantified using a BCA assay kit (Beyotime, Shanghai, China).

Protein samples were subjected to enzymatic digestion and mass spectrometry analysis. The generated .raw data files were processed with MaxQuant software v2.4.7.0 and searched against the UniProt database. Differentially expressed proteins (DEPs) were screened with the criteria of *p* < 0.05 and |log_2_FC| > 1. DEPs were then subjected to Gene Ontology (GO) enrichment analysis, Kyoto Encyclopedia of Genes and Genomes (KEGG) pathway analysis, and Protein–Protein Interaction (PPI) network construction. The expression levels of key DEPs were quantified by Label-Free Quantification (LFQ) intensities, and the differences in key protein expression were verified via Western blot analysis.

### 2.9. Statistical Analysis

All data were expressed as mean ± standard deviation (SD). Statistical analysis and graph plotting were performed using GraphPad Prism software v10.1.2 (GraphPad Software, San Diego, CA, USA). For comparisons between two independent samples, normally distributed data were analyzed using the Unpaired Student’s *t*-test, while non-normally distributed data were analyzed using the Mann–Whitney U test. For comparisons among three or more groups, one-way Analysis of Variance (ANOVA) followed by Tukey’s post hoc test was used. The significance levels for statistical differences were set as * *p* < 0.05, ** *p* < 0.01, and *** *p* < 0.001.

### 2.10. Utilization of AI Tools

In this study, artificial intelligence tools were employed to perform partial language translation and grammatical modification.

## 3. Results and Discussion

### 3.1. Material Characterization

**Figure 1:**
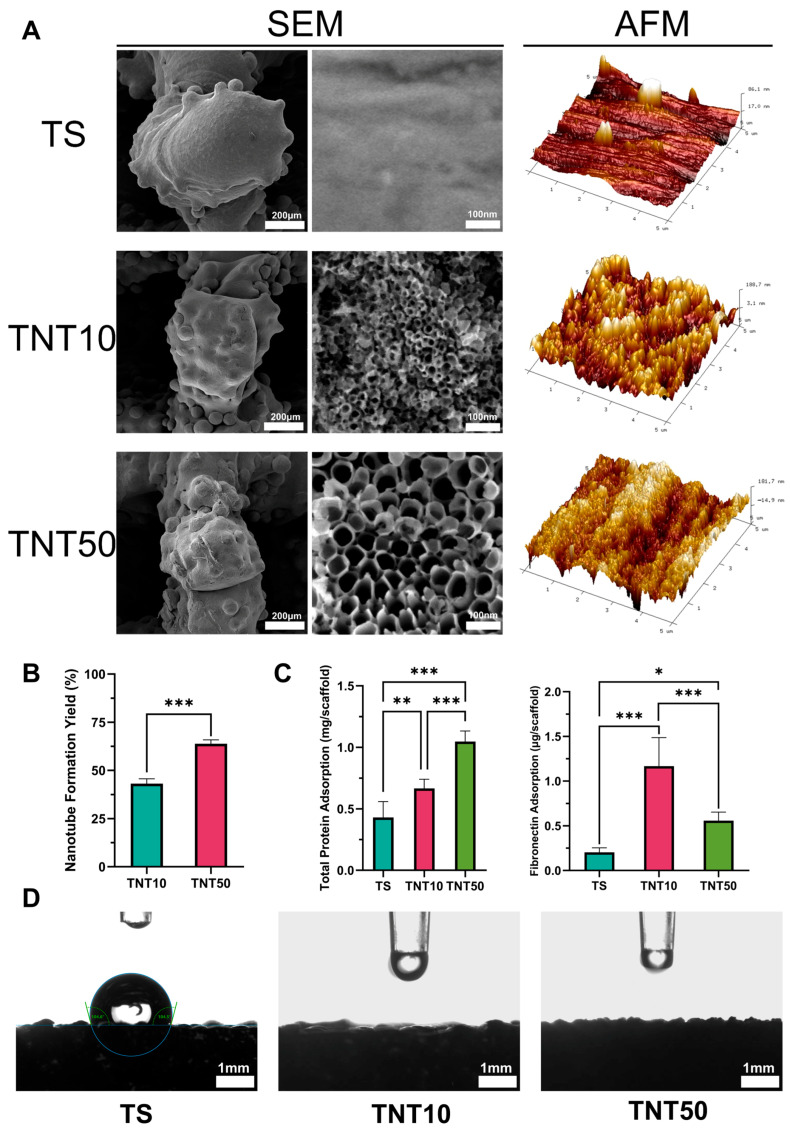
Characterization of surface morphology and physicochemical properties of the samples. (**A**) Representative SEM images and three-dimensional AFM topographic images of the samples in each group. (**B**) Quantitative analysis of nanotube formation rate under different anodizing voltages (*n* = 5). (**C**) Quantitative analysis of total protein and fibronectin adsorption on the sample surfaces (*n* = 5). (**D**) Water contact angle measurement. TNT surfaces exhibited a nearly 0° water contact angle, indicating a superhydrophilic surface after anodization. * *p* < 0.05, ** *p* < 0.01, and *** *p* < 0.001.

Highly ordered TNT arrays were successfully fabricated on Ti-6Al-4V scaffolds via electrochemical anodization (Figure 1A). AFM three-dimensional topographic analysis showed that anodization significantly increased surface roughness, as reflected by higher Ra, Rq, and SAD values, compared to the untreated TS group (Figure 1A and Table 1). As the anodization voltage increased from 10 to 50 V, the average nanotube diameter increased from 11.5 ± 5.5 nm to 65.9 ± 11.2 nm, while the nanotube length increased from 432 ± 61.9 nm to 1023 ± 85.0 nm. Meanwhile, the tubular morphology became more uniform and structurally intact. Quantitative image analysis further showed that the nanotube formation efficiency was significantly higher in the TNT50 group than in the TNT10 group (Figure 1B and Figure S1). These results indicate that anodization voltage is a key determinant of nanotube diameter, length, and growth quality, which is consistent with recent systematic and mechanistic studies showing strong voltage dependence of TiO_2_ nanotube growth during anodization [18].

As shown in Figure 1C, anodization significantly increased the total amount of adsorbed protein compared to the untreated scaffold, and total protein adsorption further increased with increasing anodization voltage. In contrast, the amount of the adhesion-related protein fibronectin was higher in the low-voltage group than in the high-voltage group, which may be closely related to the greater early cell adhesion observed subsequently in this group. This trend is in line with previous reports showing that TNT geometry affects not only the total amount of adsorbed serum proteins, but also the composition and bioactivity of the adsorbed protein layer, thereby influencing downstream cell adhesion behavior [13,19,20,21].

EDS analysis showed that, after anodization, the relative contents of Ti and Al decreased, whereas the O content increased markedly and the F content increased slightly (Table 2 and Figure S2). These compositional changes are mainly attributable to the formation of the TiO_2_ nanotube layer during anodization.

Regarding wettability, the original sessile-drop measurements on the untreated TSs yielded an apparent contact angle above 90° (Figure 1D and Table 1), whereas the TNT-coated samples exhibited nearly instantaneous water spreading and penetration, with an apparent contact angle approaching 0°, indicating a superhydrophilic-like wetting response. According to commonly used contact-angle classifications for flat, non-porous surfaces, water contact angles below 10° are generally considered superhydrophilic, whereas those above 90° are classified as hydrophobic [22]. Based on this convention, the TNT-coated surfaces can be described as falling within the superhydrophilic regime. Such interfacial characteristics, together with the increased nanoscale roughness, are favorable for early protein conditioning and integrin-mediated cell adhesion [23,24]. However, the present samples possess large macropores and an open three-dimensional lattice architecture. Under these conditions, the measured contact angle is strongly affected by droplet bridging, liquid penetration, and pore geometry, rather than intrinsic surface wettability alone. Therefore, the measured values should be interpreted as reflecting apparent wetting behavior rather than intrinsic surface wettability, and a direct wettability comparison between TS and TNT-coated porous scaffolds is methodologically limited.

### 3.2. Cell Adhesion and Proliferation

**Figure 2:**
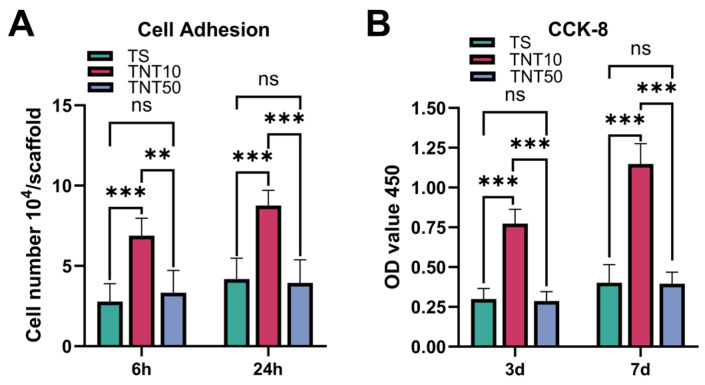
Quantitative evaluation of adhesion and proliferation behaviors of hBMSCs on surfaces of different samples. (**A**) Number of adherent hBMSCs on each group after 6 and 24 h of culture (*n* = 5). (**B**) CCK-8 assay of hBMSC proliferation on days 3 and 7. ns *p* > 0.05 * *p* < 0.05, ** *p* < 0.01, and *** *p* < 0.001.

Quantitative analysis of cell adhesion showed that TNT topography exerted a clear size-dependent effect on hBMSC adhesion (Figure 2A). Compared to the untreated TS group, the small-diameter nanotube group (TNT10) significantly increased the number of adherent hBMSCs at both 6 and 24 h. In contrast, although the TNT50 group also possessed a nanotubular architecture, the number of adherent cells decreased relative to TNT10 and was not significantly different from that of the TS group. These findings suggest that nanotube topography is a critical factor determining early cell–material interface interactions.

The CCK-8 results were consistent with the adhesion data (Figure 2B). On days 3 and 7, hBMSCs cultured on TNT10 exhibited significantly higher proliferation activity than those cultured on TS and TNT50, whereas no significant difference was observed between TNT50 and TS. Taken together, these results indicate that small-diameter TNT arrays are more favorable for the early adhesion and subsequent proliferation of hBMSCs than larger-diameter nanotubes. This further suggests that an appropriate nanoscale topography, rather than a larger nanotube diameter alone, provides a more suitable interfacial microenvironment for cell growth. This phenomenon is consistent with previous studies on the size effects of TNTs. A high-throughput study using gradient TiO_2_ nanotubes showed that, although total protein adsorption increased with nanotube diameter, cell proliferation and differentiation were preferentially supported on relatively small-diameter nanotubes (<70 nm), and the authors further noted that small-diameter TNTs were superior for focal adhesion-related cell attachment and growth [13].

In general, our findings confirm that “a larger nanotube diameter does not necessarily confer superior biological performance”. Instead, there exists an optimal size window that is favorable for hBMSC adhesion and proliferation. In this study, small-diameter nanotubes (TNT10) are more conducive to establishing stable early cell–material interactions, thus providing a foundation for subsequent osteogenesis-related biological processes.

### 3.3. Cell Morphology and Focal Adhesion Staining

**Figure 3:**
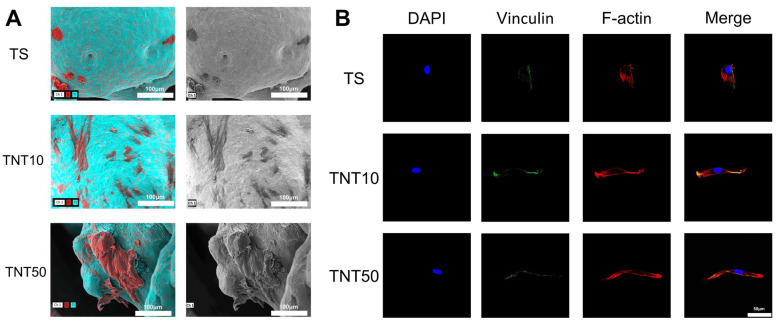
Morphological characterization of hBMSCs cultured on different sample surfaces. (**A**) Representative SEM images with EDS of hBMSCs cultured on sample surfaces. Blue indicates the Ti element, representing the titanium substrate, while red indicates the C element, which mainly corresponds to the attached hBMSCs. Cells on the TS surface exhibited a rounded morphology, whereas cells on the anodized surface were flatter and more spread out. (**B**) Representative CLSM images of hBMSCs cultured on sample surfaces. The cytoskeletal organization was more pronounced in the anodized groups, and vinculin expression was most evident in the TNT10 group.

The SEM and CLSM results showed that, compared to untreated surfaces, hBMSCs cultured on TNT surfaces exhibited a flatter and more fully spread morphology (Figure 3 and Figure S3). Cells in the TNT groups displayed a larger spreading area, more evident filopodial extension, and a more continuous and organized F-actin cytoskeleton, with the cytoskeletal organization appearing particularly pronounced in the TNT50 group. In addition, hBMSCs on TNT surfaces exhibited higher expression of the focal adhesion-associated protein vinculin, with the most prominent staining observed in the TNT10 group, especially at the distal regions of extending pseudopodia. These observations confirm that TNT surfaces can significantly promote dynamic cytoskeletal changes and cell spreading, thereby enhancing early cell–material interfacial adhesion.

Previous studies have shown that TNTs can regulate cell morphology and behavior through their nanoscale topography by modulating integrin-mediated adhesion, focal adhesion assembly, and F-actin reorganization [25,26]. Therefore, the enhanced cell spreading and improved cytoskeletal organization observed in this study are likely the critical cytological basis for TNT surfaces to promote the early adhesion of hBMSCs.

Notably, although TNT50 showed more obvious cytoskeletal extension and spreading, vinculin expression was strongest in the TNT10 group, suggesting that extensive cell spreading alone does not necessarily indicate the most favorable early adhesive state. Instead, early adhesion appears to depend on coordinated regulation of multiple processes, including protein conditioning, integrin engagement, focal adhesion stabilization, and cytoskeletal organization. This interpretation is consistent with previous reports showing that small-diameter TNTs are generally more favorable for focal adhesion-related cell attachment, whereas larger nanotubes may still support pronounced cell spreading or later-stage cytoskeletal maturation [13,27].

Mechanistically, the superior adhesion and proliferation of hBMSCs on TNT10 may be attributed to its optimal nanoscale dimensions that promote integrin recognition and focal adhesion formation [21]. A plausible explanation is that the shorter local spanning distance on small-diameter nanotube surfaces facilitates integrin clustering and the establishment of stable focal adhesion complexes. By contrast, when the nanotube diameter becomes excessively large, the increased spacing between adjacent nanotube walls may weaken effective cell anchorage and reduce early adhesion efficiency, even though cytoskeletal extension remains evident [23,28].

Taken together, these results suggest that TNT-induced regulation of hBMSC behavior is not governed by a single morphological parameter. Rather, different nanotube sizes may preferentially affect distinct aspects of cell response. In the present study, the TNT50 surface appeared to promote more pronounced cytoskeletal extension, whereas TNT10 provided a more favorable microenvironment for focal adhesion formation and stable early cell attachment, which is more consistent with the higher adhesion and proliferation observed in this group.

### 3.4. Proteomics—Mechanisms Promoting Adhesion

**Figure 4:**
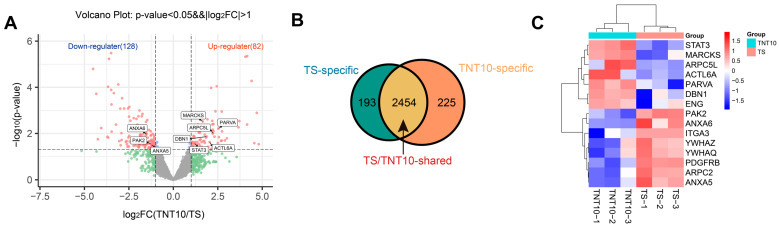
Proteomic analysis of DEPs associated with the adhesion-promoting effect of TNT10. (**A**) Volcano plot of the DEPs between the TS and TNT10 groups, with selected upregulated and downregulated proteins indicated. (**B**) Venn diagram showing shared and uniquely expressed proteins between the two groups. (**C**) Hierarchical clustering heatmap showing the expression patterns of representative DEPs across individual samples.

**Figure 5:**
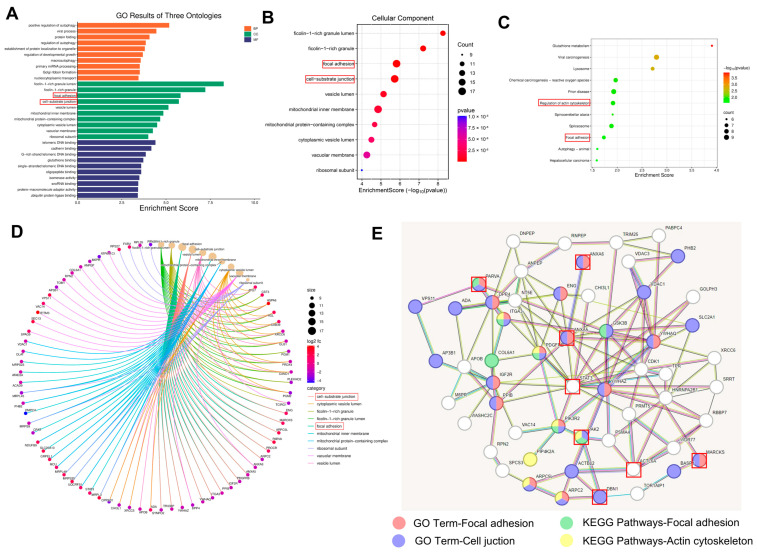
Proteomic analysis of the molecular basis of TNT10-mediated hBMSC adhesion. (**A**,**B**) GO enrichment analysis of DEPs, showing major enrichment in cellular components associated with focal adhesion and cell–substrate junctions. (**C**) KEGG enrichment bubble plot showing that focal adhesion and regulation of actin cytoskeleton were among the most significantly enriched pathways. (**D**) Chord plot illustrating the relationships between selected GO terms and their related DEPs. (**E**) PPI network showing the interactions among representative pathways and core DEPs.

To explore the molecular basis of TNT topography regulating hBMSC adhesion, proteomic analysis was performed on hBMSCs cultured on TS and TNT10. Obvious differences were observed in the protein expression profiles between the two groups (Figure 4). GO and KEGG enrichment analyses further showed that the DEPs were mainly enriched in biological modules closely related to cell–matrix interaction and mechanoadaptive responses, including focal adhesion, cell–substrate junction, and regulation of the actin cytoskeleton (Figure 5A–C). These enrichment results do not by themselves demonstrate causality, but they do indicate that TNT10 is associated with coordinated remodeling of adhesion- and cytoskeleton-related molecular programs rather than isolated changes in a few individual proteins. This interpretation is consistent with the broader view that integrin-based adhesions act as mechanoresponsive signaling hubs linking extracellular topography to actin organization and force transmission [15].

**Figure 6:**
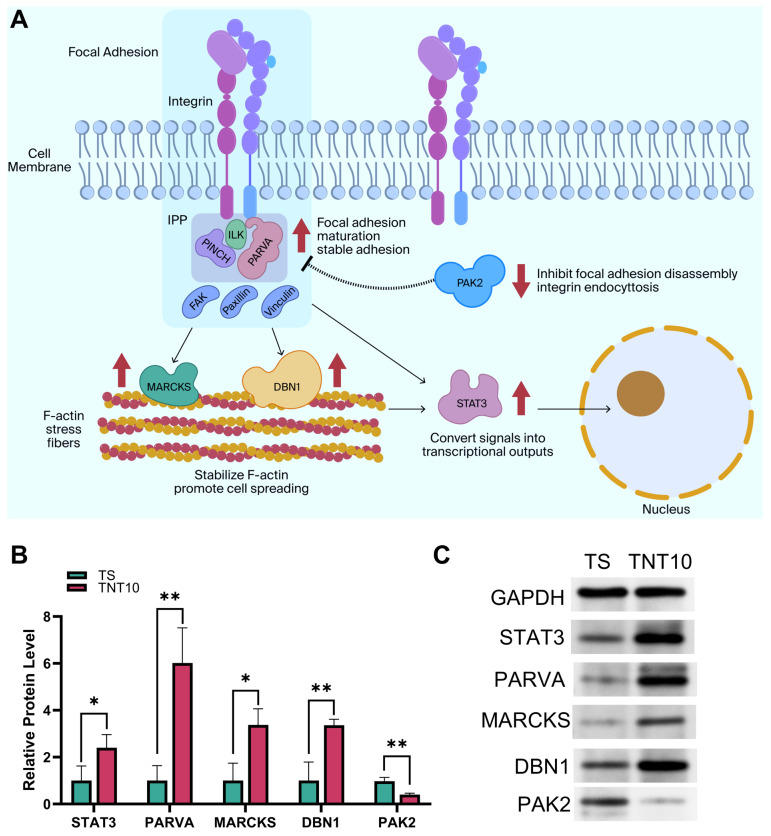
Key DEPs and the adhesion-promoting mechanism of TNT10. (**A**) Schematic diagram of the proposed mechanism by which TNT10 enhances cell adhesion. (**B**) Relative expression levels of selected key upregulated and downregulated proteins (*n* = 3). (**C**) Western blot validation of representative critical DEPs. * *p* < 0.05, ** *p* < 0.01, and *** *p* < 0.001.

After integrating the Chord plot and PPI network analyses (Figure 5D,E), several representative hub proteins, including alpha-parvin (PARVA), myristoylated alanine-rich C-kinase substrate (MARCKS), drebrin 1 (DBN1), signal transducers and activators of transcription 3 (STAT3), and p21-activated kinase 2 (PAK2), were identified and further examined by targeted quantification and Western blotting (Figure 6B,C). Importantly, these data should be interpreted as correlative molecular evidence associated with the adhesion-promoting phenotype of TNT10, rather than as definitive proof of a single causal pathway. Within this framework, our data support a working model in which TNT10 promotes the transition of hBMSCs from initial contact to more stable adhesion through coordinated regulation of adhesive protein conditioning, focal adhesion organization, and actin cytoskeletal remodeling. This interpretation is broadly consistent with previous TNT studies showing that smaller nanotube dimensions are often more favorable for early cell attachment and adhesion-related responses, whereas larger nanotubes may still support marked cytoskeletal rearrangement or spreading without necessarily producing the strongest stable adhesion phenotype [23,29].

With regard to the specific proteins highlighted by the proteomics analysis, the upregulation of PARVA is notable because it is a core component of the ILK–PINCH–parvin (IPP) complex, which functions at integrin adhesions as a structural and signaling bridge between integrins and the actin cytoskeleton [30]. Studies have shown that the IPP complex is localized at integrin adhesions and plays a vital role in focal adhesion maturation and integrin–cytoskeleton coupling [31]. Therefore, increased PARVA expression in TNT10 is compatible with strengthened integrin–cytoskeleton coupling and focal adhesion maturation.

Meanwhile, the upregulation of MARCKS and DBN1 is compatible with enhanced cortical actin remodeling and stabilization. MARCKS is a membrane-associated regulator of actin organization and cell spreading [32], whereas drebrin-family proteins such as DBN1 can bind and stabilize actin filaments and have been linked to stronger cell–substratum adhesions [33]. In this context, their increased abundance in TNT10 is consistent with the more organized cytoskeletal phenotype observed by CLSM.

In addition, the upregulation of STAT3 suggests that local mechanical/adhesive signals may be further amplified and converted into sustained transcriptional outputs. Previous studies have shown that activated STAT3 not only localizes to focal adhesion regions, but also directly regulates the expression of actin-bundling molecules such as fascin [34]. Thus, its upregulation may help convert early mechanical stimuli into stable adhesive and cytoskeletal remodeling phenotypes.

Focal adhesions are dynamic structures whose biological effect depends not only on assembly, but also on the rate of disassembly and turnover. PAK family signaling is closely involved in cytoskeletal dynamics and focal adhesion turnover. In many systems, reduced PAK/PAK2 signaling is associated with decreased migratory activity and altered focal adhesion disassembly dynamics [35,36]. In this regard, reduced PAK2 expression in TNT10 may be compatible with a shift away from a more dynamic, migration-prone state toward a more stable adhesive state.

In summary, the present data support an interpretation: TNT10 is associated with an adhesion-favoring molecular program involving focal adhesion-related, IPP-associated, and actin-regulatory proteins. Instead, the proteomics results provide a set of mechanistically plausible candidates that help explain why TNT10, but not TS, supported stronger early hBMSC adhesion.

**Figure 7:**
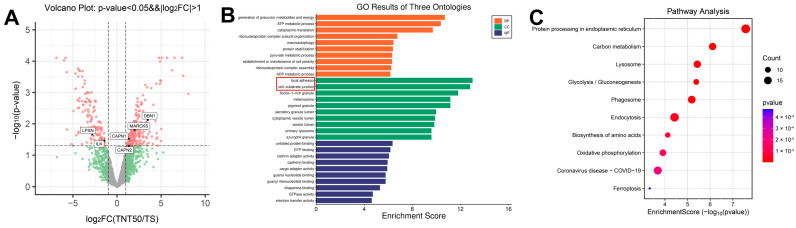
Proteomic analysis of the molecular mechanisms associated with hBMSC response to TNT50 versus TS. (**A**) Volcano plot showing the DEPs between the TS and TNT10 groups, with key upregulated and downregulated proteins labeled in the figure. (**B**) GO enrichment analysis showing that the DEPs were similarly enriched in focal adhesion- and cell–substrate junction-related terms. (**C**) Bubble plot for KEGG enrichment analysis of DEPs.

To further explore why TNT50, despite also possessing a nanotubular coating, did not enhance cell adhesion, we additionally performed proteomic analysis comparing the TNT50 and TS groups. As shown in Figure 7, MARCKS and DBN1, which were also upregulated in TNT10 and are associated with actin organization and stabilization, were likewise significantly upregulated in TNT50. This finding is consistent with the relatively pronounced actin cytoskeletal structures observed in the TNT50 group by CLSM, suggesting that TNT50 is still capable of promoting cytoskeletal remodeling to some extent. Although enrichment analysis again identified focal adhesion- and cell–substrate junction-related terms, closer examination of the differentially expressed proteins suggested that the adhesive phenotype of TNT50 may differ from that of TNT10, with a shift from more stable adhesion toward a more dynamic, higher-turnover adhesion state.

Among the altered proteins, integrin-linked kinase (ILK) is particularly noteworthy because it is a core scaffold component of integrin adhesion complexes and, together with PINCH and PARVA, forms the IPP complex that links integrins to the actin cytoskeleton and supports focal adhesion organization and maturation. Therefore, the downregulation of ILK in TNT50 is compatible with reduced stability of cell–matrix adhesion structures and weaker integrin–cytoskeleton coupling [30,37]. In addition, leupaxin (LPXN), a paxillin-family focal adhesion adaptor, participates in adhesion signaling, cell spreading, and migration-related regulation. Existing evidence indicates that leupaxin contributes to adhesion-zone organization and can modulate integrin-dependent adhesion events; accordingly, its downregulation in TNT50 further supports the possibility that mature adhesion complex organization and associated signal integration are weakened in this group [38,39]. By contrast, the upregulation of CAPN1/2 (calpain-1/2) suggests increased focal adhesion turnover. Calpain is well known to promote adhesion disassembly through limited proteolysis of focal adhesion proteins, and talin is a particularly important calpain substrate in this process. Previous studies have shown that calpain-mediated talin cleavage is a rate-limiting step in focal adhesion turnover and is critical for adhesion disassembly [40,41]. Therefore, increased CAPN1/2 expression in TNT50 is more consistent with enhanced adhesion remodeling than with reinforced static adhesion.

Taken together, these molecular changes are more compatible with a phenotype characterized by reduced stable attachment and enhanced adhesion dynamics, rather than simply weaker adhesion in a static sense. In other words, TNT50 may still support marked cytoskeletal extension, but the associated adhesion structures appear more likely to remain in a relatively dynamic remodeling state, which is generally more favorable for migration-related behavior than for stable early attachment. This interpretation also helps explain why obvious actin rearrangement could still be observed in TNT50 despite its failure to improve early adhesion and proliferation to the same extent as TNT10.

This study has several limitations. First, the proteomic findings provide correlative rather than causal evidence, and therefore the causal relationships among key molecular nodes still need further verification through siRNA interference, inhibitor treatment, or co-immunoprecipitation experiments. Second, all current data were obtained from in vitro experiments, and thus, the relevance of these findings to the in vivo microenvironment remains to be established. At last, although the nanotube diameter was the most prominent difference between the TNT10 and TNT50 groups, other surface characteristics also changed to some extent with anodization voltage, including nanotube length and subtle variations in surface elemental composition. Therefore, the present study cannot conclude that all observed biological effects were exclusively caused by nanotube diameter alone, and the potential combined contributions of other physicochemical factors should be considered in future investigations.

## 4. Conclusions

In this study, ordered TiO_2_ nanotube arrays with distinct nanoscale dimensions were successfully fabricated on porous Ti-6Al-4V scaffolds by anodization, and their effects on hBMSC adhesion behavior were systematically evaluated. Compared to the untreated scaffold and the large-diameter TNT50 surface, the small-diameter TNT10 surface more effectively promoted hBMSC adhesion and proliferation. These results indicate that an appropriate nanotopographical size window, rather than a larger nanotube diameter alone, is more favorable for establishing stable early cell–material interactions.

Proteomic analysis further showed that TNT10 was associated with coordinated changes in focal adhesion-, IPP complex-, and actin cytoskeleton-related proteins, suggesting the presence of an adhesion-favoring molecular program. In contrast, TNT50, despite still inducing obvious cytoskeletal remodeling, appeared to be more compatible with a relatively dynamic, higher-turnover adhesion state rather than a stable adhesive phenotype. Together, these findings help explain why different TNT dimensions can produce divergent biological responses even when both surfaces possess nanotubular structures.

The present work supports the view that nanotube dimension is an important regulator of hBMSC interfacial behavior and that small-diameter TNTs are more advantageous for promoting stable adhesion on porous titanium scaffolds. At the same time, the proteomic results should be interpreted as providing mechanistically plausible candidates rather than definitive causal proof. In conclusion, this study clarifies the molecular mechanisms of TNT topography regulating cell adhesion and cytoskeletal remodeling from a proteomic perspective, providing important theoretical insights for the rational design and functional optimization of orthopedic implant interfaces.

## Figures and Tables

**Table 1:** Physicochemical properties of the samples.

Parameter	TS	TNT10	TNT50	Significance
Surface roughness				
Ra (nm)	34.1 ± 9.2	47.8 ± 5.0	61.2 ± 13.4	* *p* < 0.05
Rq (nm)	44.2 ± 11.7	59.6 ± 5.7	76.5 ± 13.8	* *p* < 0.05
SAD (%)	2.6 ± 1.6	22.2 ± 0.8	28.0 ± 6.1	*** *p* < 0.001
Static water contact angle (°)	104.2 ± 1.0	0	0	*** *p* < 0.001
Nanotube diameter (nm)	/	11.5 ± 5.5	65.9 ± 11.2	*** *p* < 0.001
Nanotube length (nm)	/	432 ± 61.9	1023 ± 85.0	*** *p* < 0.001

* *p* < 0.05, ** *p* < 0.01, and *** *p* < 0.001.

**Table 2:** Surface elemental composition of the different samples (atomic %).

Sample	Ti	O	Al	V	F	C
TS	64.9	13.5	9.0	2.4	0.4	9.6
TNT10	60.3	23.4	4.8	2.5	3.9	4.8
TNT50	44.2	32.7	5.5	2.1	5.8	5.9

## Data Availability

The authors declare that all data supporting the findings of this study are available within this paper and its Supplementary Files. The proteomics data generated in this study have been deposited to the ProteomeXchange database under accession number PXD072594. Source data are provided with this paper.

## Supplementary Materials

The following supporting information can be downloaded at: https://www.mdpi.com/article/10.3390/jfb17040200/s1, Figure S1: Schematic diagram of automatic identification of nanotubes based on Amira software; Figure S2: Surface elemental composition of the different samples determined via EDS; Figure S3: Representative CLSM images of hBMSCs cultured on sample surfaces (low-power view).

## Abbreviations

The following abbreviations are used in this manuscript:

AFM	atomic force microscope
ANOVA	analysis of variance
BCA	bicinchoninic acid
CCK-8	cell counting kit-8
CLSM	confocal laser scanning microscope
DBN1	drebrin 1
DEPs	differentially expressed proteins
EDS	energy-dispersive X-ray spectroscopy
FBS	fetal bovine serum
FN	fibronectin
GO	Gene Ontology
hBMSC	human bone marrow mesenchymal stem cell
ILK	integrin-linked kinase
IPP	ILK–PINCH–parvin
KEGG	Kyoto Encyclopedia of Genes and Genomes
LFQ	Label-Free Quantification
LPXN	leupaxin
MARCKS	Myristoylated alanine-rich C-kinase substrate
OD	optical density
PAK2	p21-activated kinase 2
PARVA	alpha-parvin
PBS	phosphate-buffered saline
PPI	protein–protein interaction
Ra	arithmetic mean roughness
Rq	root mean square roughness
SAD	surface area difference
SD	standard deviation
SEM	scanning electron microscope
STAT3	signal transducers and activators of transcription 3
TNT	titanium dioxide nanotube
TS	titanium alloy scaffold
