# Supplementary material for: Nanotopography-Mediated Mechanotransduction Enhances hBMSCs Adhesion on TiO2 Nanotubes
Source: J Funct Biomater. 2026 Apr 19;17(4):200. doi: 10.3390/jfb17040200 (PMC13118223; doi:10.3390/jfb17040200)
Supplement: Supplementary file 1 [file jfb-17-00200-s001.zip › jfb-4236090-supplementary.pdf]

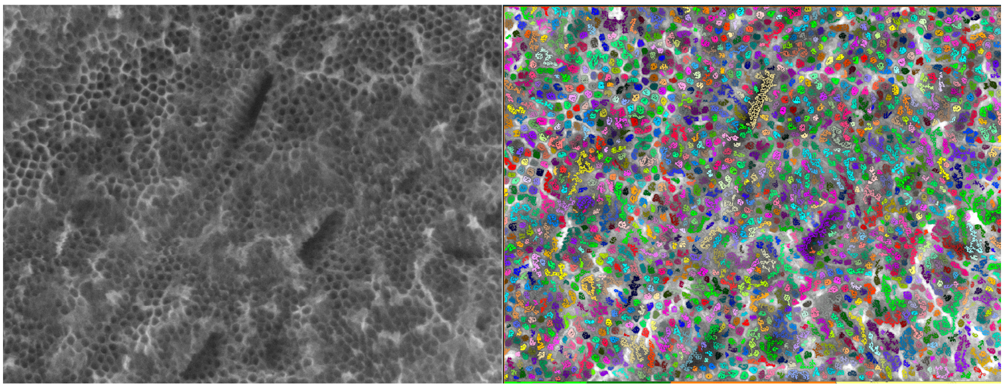

Figure S1. Schematic diagram of automatic identification of nanotubes based on Amira software.

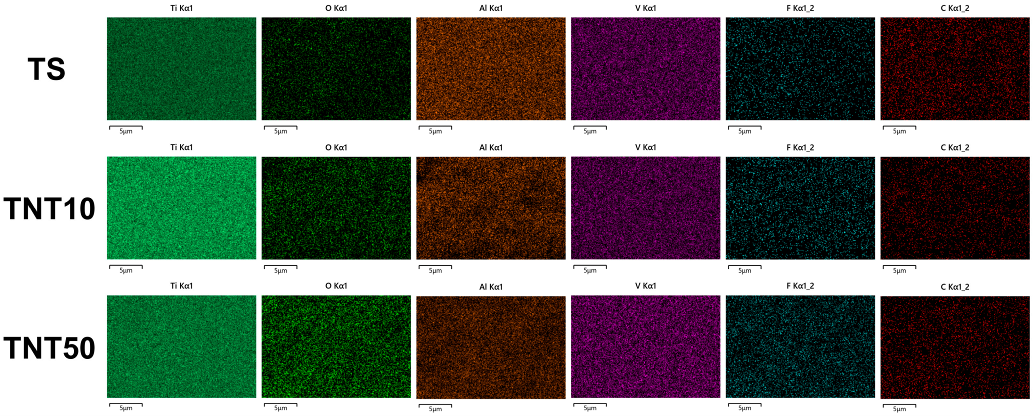

Figure S2. Surface elemental composition of the different samples determined via EDS.

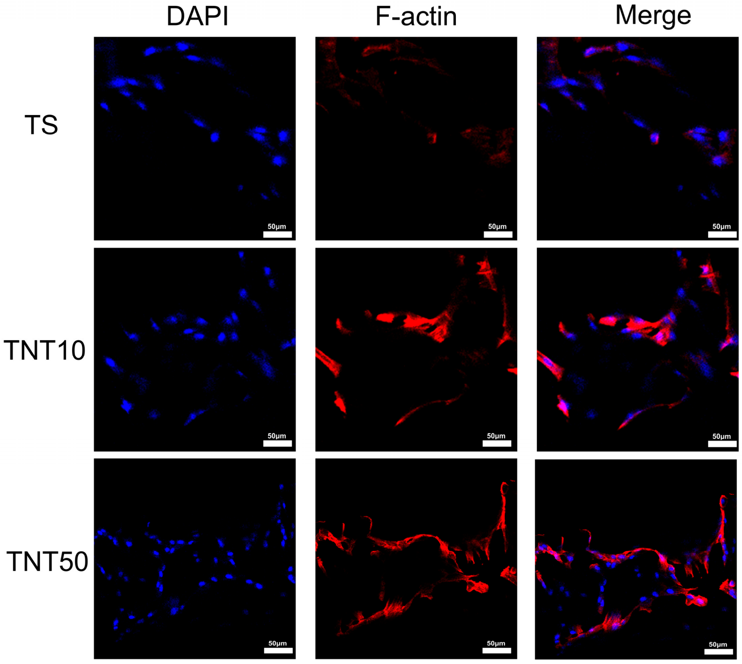

Figure S3. Representative CLSM images of hBMSCs cultured on sample surfaces (low-power view).
